# Elevated interleukin-8 enhances prefrontal synaptic transmission in mice with persistent inflammatory pain

**DOI:** 10.1186/1744-8069-8-11

**Published:** 2012-02-12

**Authors:** Guang-bin Cui, Jia-ze An, Nan Zhang, Ming-gao Zhao, Shui-bing Liu, Jun Yi

**Affiliations:** 1 Department of Diagnostic Radiology, Tangdu Hospital, Xi'an 710032, China; 2 Department of General Surgery, Xijing Hospital, Fourth Military Medical University, Xi'an 710032, China; 3 Department of Pharmacology, Fourth Military Medical University, Xi'an 710032, China

**Keywords:** Interleukin-8, Inflammation, Pain, Cingulate cortex

## Abstract

**Background:**

Interleukin-8 (IL-8) is known for its roles in inflammation and plays critical roles in the development of pain. Its expression increases in the brain after peripheral inflammation. Prefrontal cortex, including the anterior cingulate cortex (ACC), is a forebrain structure known for its roles in pain transmission and modulation. Painful stimuli potentiate the prefrontal synaptic transmission, however, little is known about the expression of IL-8 and its role in the enhanced ACC synaptic transmission in animals with persistent inflammatory pain.

**Findings:**

In the present study, we examined IL-8 expression in the ACC, somatosensory cortex (SSC), and the dorsal horn of lumbar spinal cord following hind-paw administration of complete Freund's adjuvant (CFA) in mice and its effects on the ACC synaptic transmission. Quantification of IL-8 at protein level (by ELISA) revealed enhanced expression in the ACC and spinal cord during the chronic phases of CFA-induced peripheral inflammation. In vitro whole-cell patch-clamp recordings revealed that IL-8 significantly enhanced synaptic transmission through increased probability of neurotransmitter release in the ACC slice. ACC local infusion of repertaxin, a non-competitive allosteric blocker of IL-8 receptors, notably prolonged the paw withdrawal latency to thermal radian heat stimuli bilaterally in mice.

**Conclusions:**

Our findings suggest that up-regulation of IL-8 in the ACC partly attributable to the enhanced prefrontal synaptic transmission in the mice with persistent inflammatory pain.

## Findings

Chemokines, a family of proinflammatory cytokines play a role in immune system regulation, cell growth, cell development, and inflammation [1]. Interleukin-8 (IL-8), also known as CXCL8, is an α chemokine and its main function is chemotaxis of neutrophils and T lymphocytes [2]. In the immune system, IL-8 exerts its biological action by binding to seven-transmembrane G-protein-coupled receptors named CXCR1 and CXCR2. In the CNS, CXCR2 receptors have been described on astrocytes, microglia, and neurons [3]. The chemokine IL-8 is known to be synthesized by microglial cells and astrocytes [4]. IL-8 may be an important factor in intercellular communication between glia and neurons by rapidly altering the excitability of neurons, probably through presynaptic mechanisms [5]. Repertaxin is a new non-competitive allosteric blocker of IL-8 receptors (CXCR1/R2), which by locking CXCR1/R2 in an inactive conformation prevents receptor signaling and human polymorphonuclear leukocyte (PMN) chemotaxis [6].

Studies using different experimental approaches consistently suggest that the anterior cingulate cortex (ACC) plays important roles in processing pain-related information in humans and in the behavioral responses to noxious stimuli or tissue injury in animals [7]. Our previous studies show that synaptic transmission in the ACC is enhanced in mice with persistent inflammatory pain induced by hind-paw injection of complete Freund's adjuvant (CFA) [8-10]. According to current concepts, cytokines in the CNS are likely to behave as neuromodulators. Recent studies have demonstrated that IL-8 and other chemokines elicit rapid signaling events in neurons to modulate excitatory and inhibitory synaptic activities [11]. It has been implicated in chronic inflammatory pain states [12]. Evidence shows that IL-8 expression increases in the spinal dorsal horn in a rat model of lumbar disc herniation [13]. The pathophysiological condition, such as traumatic brain injuries, induces local brain and systemic stress response, in which IL-8 is considered as a key mediator of neuroinflammation [14]. Our previous studies show that synaptic transmission in the ACC is enhanced in mice with persistent inflammatory pain induced by hind-paw injection of CFA, and presynaptic alterations caused by peripheral inflammation is partly attributable to the up-regulation of tumor necrosis factor alpha (TNF-α) in the ACC [8,9]. The synergistic effect on IL-8 secretion of TNF-α has been implicated [15]. However, it is not clear what is the role of IL-8 in the enhanced ACC synaptic transmission in animals with persistent inflammatory pain. To address this question, we performed experiments to quantify the IL-8 expression and identify its roles in ACC synaptic transmission and pain modulation after hind-paw inflammation in vitro and in vivo.

**Figure 1:**
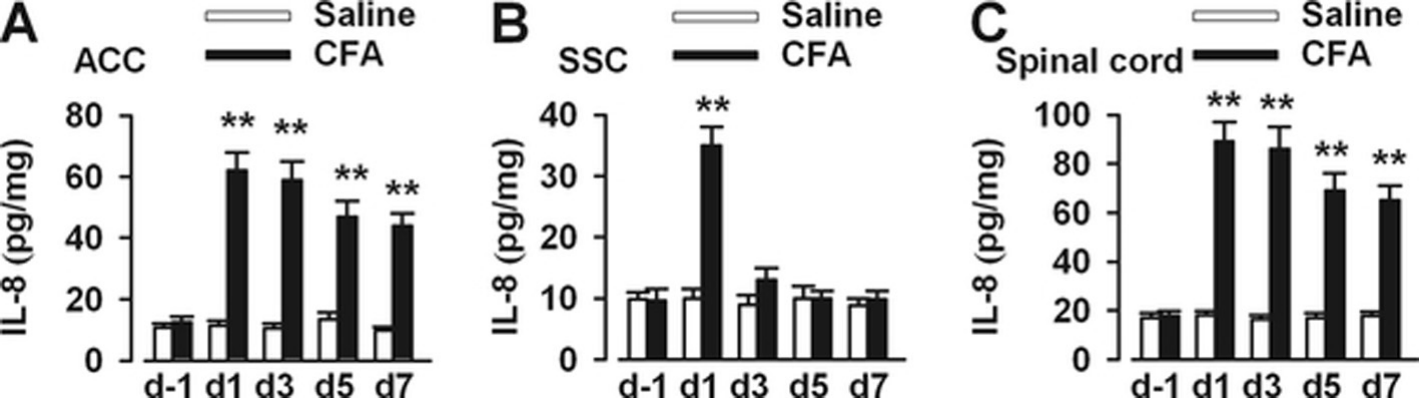
**Levels of IL-8 in the brain following hind-paw CFA injection**. **(A) **There was a significant increase in the IL-8 concentration in the ACC at d1, d3, d5, and d7 after CFA injection compared with saline injection (*n *= 6 in each time point). **(B) **There was a significant increase in the IL-8 concentration in the SSC at d1 but no difference at d3, d5, and d7 after CFA injection compared with saline injection (*n *= 6 in each time point). **(C) **There was a significant increase in the IL-8 concentration in the dorsal horn of lumbar spinal cord at d1, d3, d5, and d7 after CFA injection compared with saline injection (*n *= 6 in each time point). ** *P *< 0.01 compared with saline controls.

To test whether the IL-8 was involved in enhanced synaptic transmission in the ACC after chronic pain, we measured IL-8 concentration in ACC at 1, 3, 5, and 7 days after hind-paw injection of CFA. Increased expression of IL-8 in the ACC was seen at day 1 following peripheral administration of CFA (the acute phase of inflammation) and remained elevated during the chronic phases (day3-day7) of inflammation (Figure 1A). To rule out the possibility that increased expression of IL-8 is a nonspecific result of chronic pain, not limited to changes in cingulate cortex, we tested IL-8 in the somatosensory cortex (SSC) of the same animals. As shown in Figure 1B, expression of IL-8 in the SSC was increased at day 1 following peripheral administration of CFA and decreased to the basal level during the chronic phases (day 3-day7) (Figure 1B). Last, we also tested IL-8 in the dorsal horn of lumbar spinal cord in the same animals. Expression of IL-8 was increased following peripheral administration of CFA (day 1-day7) (Figure 1C). These results suggest that increased expression of IL-8 is regionally specific to the ACC and dorsal horn of lumbar spinal cord during the chronic phases of hind-paw inflammation.

**Figure 2:**
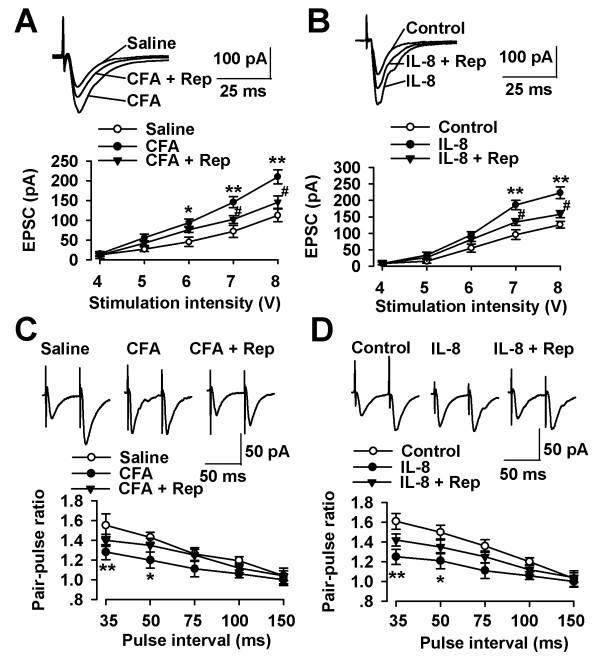
**Potentiation of synaptic transmission in the ACC slices by IL-8**. **(A) **Representative recordings of evoked EPSCs in the ACC slices from saline, CFA-injected, and ACC infusion of repertaxin mice (upper). Plot of input-output curves showed the enhancement of synaptic transmission in the ACC of CFA-injected mice and ACC infusion of repertaxin partially inhibited CFA-induced synaptic transmission. *n *= 9 slices/4 mice in each group, * *P *< 0.05, ** *P *< 0.01 compared with saline-injected mice; ^# ^*P *< 0.05 compared with CFA-injected mice. **(B) **Representative recordings of evoked EPSCs in the control and IL-8 incubated ACC slices (upper). Plot of input-output curves showed enhancement of synaptic transmission by IL-8 perfusion; however, ACC infusion of repertaxin partially inhibited IL-8-induced synaptic transmission. *n *= 8 slices/4 mice in each group, * *P *< 0.05, ** *P *< 0.01 compared with control; ^# ^*P *< 0.05 compared with IL-8 incubated slices. **(C) **Representative traces of PPF with an interval of 50 ms recorded in the saline, CFA-injected, and ACC infusion of repertaxin mice (upper). PPF was notably reduced in CFA-injected mice, and ACC infusion of repertaxin reversed the reduced PPF in the ACC of CFA-injected mice. *n *= 10 slices/4 mice in each group, * *P *< 0.05, ** *P *< 0.01 compared with saline-injected mice. **(D) **Representative recordings of PPF in the control and IL-8 incubated ACC slices (upper). PPF was significantly reduced by IL-8 incubated slices, and ACC infusion of repertaxin reversed the reduced PPF in the ACC of IL-8 incubated slices. *n *= 9 slices/4 mice in each group, *P < 0.05, ** *P *< 0.01 compared with control.

The present result shows that the increment time course of IL-8 amount in the ACC is consistent with the time window of enhanced synaptic transmission with persistent inflammatory pain, raising the possibility of IL-8 playing a role in the modulation of ACC synaptic transmission. To test this hypothesis, excitatory postsynaptic currents (EPSCs) from layer II-III neurons were elicited by a bipolar tungsten-stimulating electrode placed in layer V of the ACC. The input-output relationships, measuring EPSCs amplitude (output) as a function of the afferent stimulus intensity (input), were compared as described [8]. Synaptic transmission in the ACC was significantly enhanced in the slices of mice after hind-paw CFA injection within day 3 to day 7 (Figure 2A and 2B). ACC local infusion of repertaxin (0.5 μg/0.5 μl), a non-competitive allosteric blocker of IL-8 receptors, notably reversed the enhanced synaptic transmissions in the mice with hind-paw CFA injection (Figure 2A). Furthermore, bath application IL-8 (50 ng/ml, 4 h) significantly increased the synaptic transmission in the slices from the control mice (Figure 2B). This result is consistent with the idea that persistent inflammation leads to an increase of endogenous IL-8 that shares one or more steps with exogenous IL-8.

Next, to test whether presynaptic mechanisms contributes to altered synaptic transmission by IL-8, we measured paired-pulse facilitation (PPF, the ratio of EPSC2/EPSC1) in neurons from the ACC. PPF was recorded with intervals of 35, 50, 75, 100, and 150 ms. PPF has been used as a tool to implicate presynaptic probability of transmitter release [16]. PPF was significantly decreased in ACC slices from the mice with hind-paw CFA injection and ACC local infusion of repertaxin abolished the alteration of PPF (Figure 2C). In addition, incubation of exogenous IL-8 (50 ng/ml) for 4 h also reduced the PPF in the slices from control mice (Figure 2D), indicating an increased presynaptic probability of transmitter release by IL-8.

**Figure 3:**
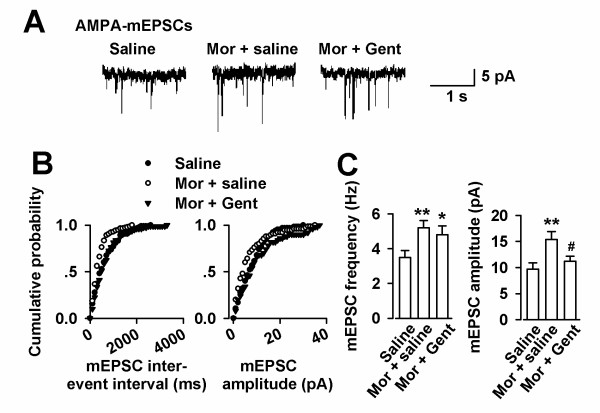
**Effects of repertaxin on basal glutamatergic synaptic transmission**. **(A) **mEPSCs recorded a holding potential of -70 mV in pyramidal neurons from saline, CFA-injected, and ACC infusion of repertaxin mice. **(B) **Cumulative frequency (left) and amplitude (right) histogram of the mEPSCs in neurons from saline, CFA-injected, and ACC infusion of repertaxin mice. **(C) **Summery of mEPSCs frequency (left) and amplitude (right) in neurons from saline (n = 10 neurons/4 mice), CFA-injected (n = 11 neurons/4 mice), and repertaxin (n = 12 neurons/4 mice) infusion mice. ** *p *< 0.01 compared with saline-injected mice; ^# ^*p *< 0.05 compared to the CFA-injected mice.

To further determine the presynaptic or postsynaptic component associated with an increase of the synaptic transmission, we recorded AMPA receptor mediated miniature excitatory postsynaptic currents (mEPSCs) in the ACC slices (Figure 3A). A significant increase in mEPSC frequency and amplitude was detected in CFA-injected mice as compared to saline controls (Figure 3B and 3C). ACC local infusion of repertaxin abolished the alteration of mEPSC frequency and amplitude (Figure 3B and 3C). These results indicate that up-regulation of excitatory synaptic transmission by IL-8 is likely due to both presynaptic transmitter release and postsynaptic AMPA receptor modifications in the ACC synapses.

**Figure 4:**
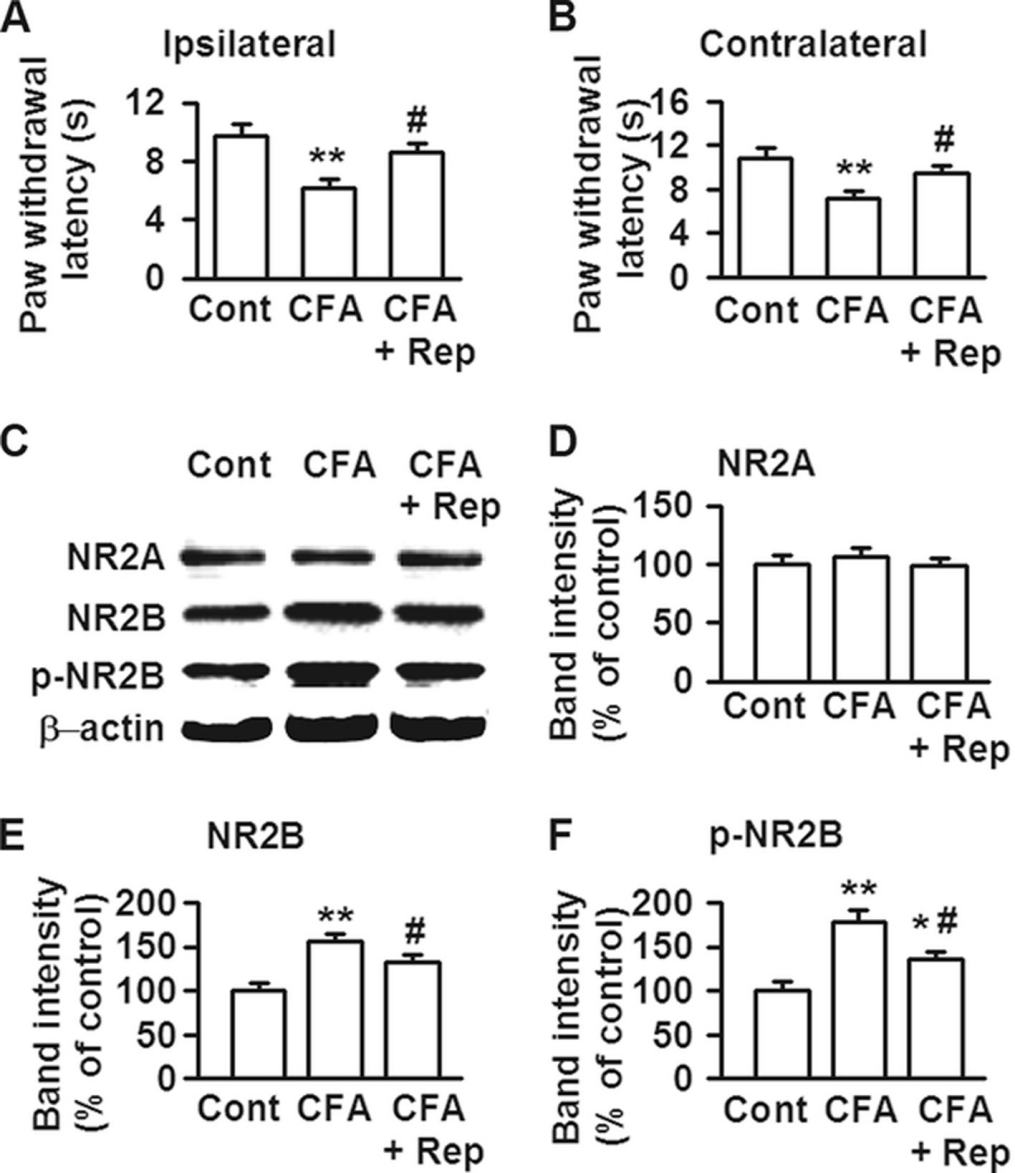
**Changes of thermal hyperalgesia and NR2B expression by IL-8**. **(A) **Compared with left paws of control, CFA-treated ipsilateral paws (left) showed a significant decrease in PWL; however, microinjection of repertaxin (0.5 μg/0.5 μl) into the ACC induced a significant increase in PWL in CFA-treated ipsilateral hind-paws. **(B) **Compared to right paws of control, CFA-treated contralateral paws (right) also showed a notably decrease in PWL, and microinjection of repertaxin (0.5 μg/0.5 μl) into the ACC induced a significant increase in PWL in CFA-treated contralateral hind-paws. *n *= 6 in each group. ** *p *< 0.01 compared with saline control; ^# ^*p *< 0.05 compared with CFA-injected alone. **(C) **Western blot analysis was performed using antibodies for NR2A, NR2B, and phosphorylated NR2B at Ser-1303 (p-Ser1303-NR2B). **(D) **There was no difference in NR2A expression among three groups. **(E-F) **NR2B and phosphorylated NR2B expressions in the ACC were notably increased at 3 d after CFA-injection; however, microinjection of repertaxin (0.5 μg/0.5 μl) into the ACC induced a significant decrease in NR2B or phosphorylated NR2B expression in CFA-injected mice. *n *= 6 in each group. * *p *< 0.05, ** *p *< 0.01 compared with control; ^# ^*p *< 0.05 compared with CFA-injected alone.

To further evaluate the role of IL-8 in the pain procedure, we detected the thermal hyperalgesia in the mice with ACC local infusion of repertaxin. The paw withdrawal latency (PWL) to thermal radian heat stimuli is a widely used nociceptive measure to study hyperalgesic behavior. As illustrated in Figure 4, CFA-treated ipsilateral paws (left) showed a significant decrease in PWL when compared to the control mice (Figure 4A). Interestingly, PWL in contralateral paws (right) also showed a significant decrease when compared to right paws of control mice (Figure 4B). Microinjection of repertaxin (0.5 μg/0.5 μl) into the ACC induced a significant increase in PWL in CFA-treated ipsilateral and contralateral hind-paws (Figure 4A and 4B).

NR2B containing NMDA receptors in the ACC play a critical role in the pain procedure [17]. Western blot results showed that both of NR2B and phosphorylated NR2B expressions in the ACC were notably increased at 3 d after CFA-injection (Figure 4C, E-F). However, no significant alteration was detected in the NR2A expression in the ACC after injury (Figure 4D). Microinjection of repertaxin (0.5 μg/0.5 μl) into the ACC induced a significant decrease in NR2B or phosphorylated NR2B expression in CFA-injected mice (Figure 4C-F). These findings imply that changes in NR2B containing NMDARs are highly related to the expression of IL-8 in the ACC.

In the present study, we found that IL-8 expression was increased specifically in the ACC and might be involved in the enhanced synaptic transmission after persistent inflammatory pain. CFA-induced peripheral inflammation induces robust glial activation and proinflammatory cytokines release including IL-8 at CNS, supporting a role of enhanced IL-8 expression at CNS in eliciting behavioral hypersensitivity [18]. In present study, IL-8 expression was found increase in the ACC at least lasting for one week after CFA hind-paw injection. This time course is parallel to the time window of enhanced synaptic transmission in the ACC induced by CFA injection as the previous study shown [8]. As a result, the increase of IL-8 expression in the ACC would affect normal signaling events. Since the prefrontal cortex is thought to be involved in the processes of chronic pain modulation [7,19], the increased ACC IL-8 expression strongly suggests that IL-8 is linked in some way to the processes of inflammatory pain. IL-8 is known to be synthesized by microglial cells and astrocytes [4]. Glial cells, including astrocytes and microglia, have recently been implicated in neuropathic pain [20]. These glial cells form close interactions with neurons and thus may modulate nociceptive transmission under pathological conditions. Even though microglia seems insensitive to neuronal activities, such as high-frequency stimulation or synaptic plasticity observed in the hippocampus [21], it is possible that microglia is response to the inflammatory stimuli in the pain related brain region such as the ACC. Inhibition of the thermal hyperalgesia by ACC local infusion of IL-8 receptor blocker provides the direct evidences of this possibility. Our previous studies show that TNF-α is partly attributable to the enhanced synaptic transmission in the ACC in mice with hind-paw injection of CFA [9]. TNF-α shows the synergistic effect on IL-8 secretion [15]. It is reasonable that IL-8 involves in the modulation of synaptic transmission in the ACC after CFA injection. However, we can not exclude the possibility that other cytokines may also involve in the synaptic transmission.

In the present study we found that IL-8 was increased both in spinal cord and ACC after inflammatory pain. However, in the somatosensory cortex expression of IL-8 in the SSC was increased only in the acute phase (one day following peripheral administration of CFA). It is consistent that ACC and spinal cord are the key brain regions involved in the pain central sensitization [22-24]. As shown in many other studies the inflamed ipsilateral paws show a significant decrease in PWL than control paws [25,26]. Also, the contralateral paws of CFA-injected group show a slight decrease in PWL than control paws, even though the decrease in PWL of contralateral paws is much smaller than that on the ipsilateral side [27,28]. This indicates that in chronic inflammatory pain condition the decrease in PWL to thermal stimuli is not confined only to the CFA-inoculated paws but also affects the non-inoculated contralateral paws. Consistent with this report, we find that unilateral CFA injection causes a bilateral decrease in PWL in present study. ACC infusion of IL-8 receptor blocker repertaxin leads to prolong PWL bilaterally, suggesting IL-8 in the ACC modulates the thermal hyperalgesia to peripheral inflammatory injury.

Present results provide the evidences that IL-8 may modulate glutamatergic synaptic transmission through both presynaptic and postsynaptic mechanism. This is demonstrated by decreased paired-pulse facilitation in ACC slice recordings and reduced APMA receptor-mediated EPSCs amplitude. The ACC NMDA receptor activities have a strong association with chronic pain [17]. The similarity in the IL-8 expression time course and roles of NMDA receptors in the pain process raises a possible relationship between IL-8 expression in the prefrontal cortex and NMDA receptor activity. Present results provide support for a novel role for IL-8 in the control of NR2B containing NMDA receptors.

Taken together, these results suggest that the up-regulation of IL-8 is involved in the enhanced alteration of synaptic transmission in the prefrontal cortex, and that IL-8 may play a role in the development of persistent inflammatory pain.

## Materials and methods

Experiments were carried out on C57BL/6 mice (8-10 weeks old of age). All animal protocols used were approved by the Animal Care and Use Committee of the Fourth Military Medical University. Mice were housed under a 12 h light/dark cycle with food and water provided ad libitum. To induce inflammatory pain, 10 μl of 50% CFA (Sigma, St. Louis, MO) was injected subcutaneously into the dorsal surface of one hind-paw. To measurement of IL-8 in the ACC, SSC, and the dorsal horn of lumbar spinal cord, mice were killed at various days after CFA injection by decapitation and the tissues were rapidly removed at 4°C and frozen on ice. The tissues were weighted and homogenized in ice-cold PBS using a Potter homogenizer. The homogenates were centrifuged for 10 min (5000 rpm, 4°C). 100 μl of supernatant were taken in duplicate to measure IL-8. The amounts of IL-8 in the ACC, SSC, and spinal cord prepared at various days after injection of CFA were determined using anti-mouse IL-8 ELISA Kits (R&D Systems, Minneapolis, MN, USA) according to the manufacturer's protocol. The concentration of the IL-8 was quantified as picogram antigen per milligram of protein.

For recording the synaptic transmission in the ACC slices, mice were anesthetized with halothane, and coronal brain slices (300 μm in thickness) were obtained using a vibrating slicer as previously described [8]. After recovery for least 1 h, slices were placed in the recording chamber and superfused at 2-3 ml/min with oxygenated (95% O_2 _and 5% CO_2_) artificial cerebrospinal fluid (aCSF) containing (in mM) 124 NaCl, 2.5 KCl, 2 CaCl_2_, 1 MgSO_4_, 25 NaHCO_3_, 1 NaH_2_PO_4_, 10 glucose. The slices were covered with a nylon mesh to prevent movement of the tissue. Experiments were performed in a recording chamber on the stage of an Olympus Microscope (Olympus, Japan) with infrared digital interference contrast optics for visualization of whole-cell patch clamp recording. EPSCs were recorded from layer II pyramidal neurons holding at -70 mV with an Axon 200B amplifier (Molecular Devices, Palo Alto, CA) and digitized at a sampling rate of 10 kHz. The pyramidal neurons were identified by the firing properties according to the previous study [29]. The stimulations were delivered by a bipolar tungsten stimulating electrode placed in layer V of the ACC at 0.05 Hz. Patch pipettes (3-5 MΩ) were filled with an internal solution containing (in mM) 145 K-gluconate, 5 NaCl, 1 MgCl_2_, 0.2 EGTA, 10 HEPES, 2 Mg-ATP and 0.1 Na_3_-GTP, which had an osmolarity of 280 mOsm and pH of 7.2. Experiments were performed at room temperature. Only experiments where there was less than 20% change in access resistance were included in analysis.

For the ACC infusion, mice were anesthetized under ketamine and xylazine. 24-guage guide cannulas were implanted bilaterally into the ACC (0.7 mm anterior to Bregma, ± 0.4 mm lateral from the midline, 1.7 mm beneath the surface of the skull). Mice were given at least 1 week to recover after cannula implantation. The 30-gauge injection cannula was 0.1 mm lower than the guide. For intra-ACC infusion, 0.5 μl repertaxin (4 μg/μl) or saline was delivered bilaterally within 90 s using a pump. Four hours later, thermal hyperalgesia was measured. The method of Hargreaves et al. (1988) was used to assess paw withdrawal latency (PWL) to a thermal nociceptive stimulus [26]. To assess thermal nociceptive responses, a plantar analgesia instrument (BME410A, Institute of Biological Medicine, Academy of Medical Science, China) was used. Animals were placed in individual plastic boxes and allowed to adjust to the environment for 1 h. Thermal hyperalgesia was assessed by measuring the latency of paw withdrawal in response to a radiant heat source. Mice were housed individually into Plexiglas chambers on an elevated glass platform, under which a radiant heat source was applied to the plantar surface of the hind-paw through the glass plate. The heat source was turned off when the mouse lifted the foot, allowing the measurement of time from onset of radiant heat application to withdrawal of the mouse hind-paw. This time was defined as the PWL. Both paws were tested alternately at 5 min intervals for a total of five trials. A 20 s cutoff was used to prevent tissue damage. Both hind-paws were tested independently with 10 min interval between trials.

Western blot analysis was performed as described previously [30]. Equal amount of proteins (50 μg) from the ACC were separated and electrotransferred onto PDVF membranes (Invitrogen), which were probed with antibodies for, NR2A (dilution ratio 1:200, Millipore, Billerica, MA), NR2B (dilution ratio 1:500), and phosphorylation of NR2B at Ser1303 (dilution ratio 1:1000, Millipore), and with β-actin (dilution ratio 1:5000, Millipore) as loading control. For data quantitation, band intensity was expressed relative to the loading control (β-actin). The membranes were incubated with horseradish peroxidase conjugated secondary antibodies (anti-rabbit/anti-mouse IgG for the primary antibodies), and bands were visualized using an ECL system (Perkin Elmer).

Results were expressed as mean ± SEM. Statistical comparisons were performed using one-way analysis of variance (ANOVA) using the Student-Newmann-Keuls for post-hoc comparisons. In all cases, *P *< 0.05 was considered statistically significant.

## Competing interests

The authors declare that they have no competing interests.

## Authors' contributions

GBC, JZA and NZ performed the experiments. MGZ analyzed the data. SBL and JY wrote the manuscript. All authors approved the final version of the manuscript.
